# A novel nomogram to predict the overall survival in esthesinoeroblastoma

**DOI:** 10.1186/s12885-020-07435-7

**Published:** 2020-10-14

**Authors:** Lijie Jiang, Tengjiao Lin, Yu Zhang, Wenxiang Gao, Jie Deng, Zhaofeng Xu, Xin Luo, Zhaoqi Huang, Fenghong Chen, Jianbo Shi, Yinyan Lai

**Affiliations:** 1 The Otorhinolaryngology Hospital, First Affiliated Hospital of Sun Yat-sen University, No.58 Zhongshan Er Road, Guangzhou, Guangzhou, 510080 P.R. China; 2 Sun Yat-sen University Cancer Center, Guangzhou, P.R. China

**Keywords:** Esthesioneuroblastoma, Prognosis, Nomogram, Survival

## Abstract

**Background:**

Increasing evidence indicates that the pathology and the modified Kadish system have some influence on the prognosis of esthesioneuroblastoma (ENB). However, an accurate system to combine pathology with a modified Kadish system has not been established.

**Methods:**

This study aimed to set up and evaluate a model to predict overall survival (OS) accurately in ENB, including clinical characteristics, treatment and pathological variables. We screened the information of patients with ENB between January 1, 1976, and December 30, 2016 from the National Cancer Institute Surveillance, Epidemiology, and End Results (SEER) program as a training cohort. The validation cohort consisted of patients with ENB at Sun Yat-sen University Cancer Center and The First Affiliated Hospital of Sun Yat-sen University in the same period, and 87 patients were included. The Pearson’s chi-squared test was used to assess significance of clinicopathological and demographic characteristics. We used the Cox proportional hazards model to examine univariate and multivariate analyses. The model coefficients were used to calculate the Hazard ratios (HR) with 95% confidence intervals (CI). Prognostic factors with a *p-*value < 0.05 in multivariate analysis were included in the nomogram. The concordance index (c-index) and calibration curve were used to evaluate the predictive power of the nomogram.

**Results:**

The c-index of training cohort and validation cohort are 0.737 (95% CI, 0.709 to 0.765) and 0.791 (95% CI, 0.767 to 0.815) respectively. The calibration curves revealed a good agreement between the nomogram prediction and actual observation regarding the probability of 3-year and 5-year survival. We used a nomogram to calculate the 3-year and 5-year growth probability and stratified patients into three risk groups.

**Conclusions:**

The nomogram provided the risk group information and identified mortality risk and can serve as a reference for designing a reasonable follow-up plan.

## Background

ENB is a rare sinonasal tumor, which derived from the olfactory epithelium at the top of nasal cavity and is also named olfactory neuroblastoma [1]. It was reported that approximately 6% of nasal cavity and paranasal sinus tumors were ENB [2–4]. Berger and his colleagues first described the malignant neoplasm in 1924, and ENB is known to show variable progression [5]. Dulguerov reported that the diagnosis difficulty, varying biological activity and lack of valid staging system with consensus may contribute to the variable progression in 2001 [6].

The Hyams grading system based on histological features was first described by Hyams [7]. Dulguerov also noted the possible role of histopathologic grading in predicting prognosis by meta-analysis [6]. In 2014, Saade reported that necrosis and mitosis were significant predictors of OS and progression-free survival (DFS) but not as individual parameters [8].

The staging classification of clinical data was first proposed by Kadish and his coworkers, which included three categories for ENB due to the shortcoming of the staging system [9]. Morita et al. modified the Kadish system by including group D, which includes patients with metastasis to the cervical lymph nodes or distant site [10]. In 2001, Dulguerov proposed the modified Kadish staging system, which is more similar to the TMN tumor system, and the criteria were based on magnetic resonance imaging (MRI) and computed tomography (CT). The modified Kadish system for ENB is more reliable in assessing the anatomical sites of disease [6].

Jethanamest and Nalavenkata respectively reported the modified Kadish staging system as risk factor used to predict the prognosis of patients with ENB and guide tumor management and treatment [11, 12]. Recently, the histopathology of the Hyams Grading System has been proven to affect the prognosis of ENBs and the treatment [13–17]. However, the single clinical staging system has not been shown to be an adequate predictor of outcome.

Our study aims to establish a nomogram based on clinical characteristics, treatment and pathological variables in predicting OS among patients with ENB. Finding a more suitable indicator for the prediction of ENB prognosis is critical.

## Methods

### Patient data collection

For the training cohort, histological feature code 9522 was used to identify all patients diagnosed with ENB from January 1, 1976, to December 30, 2016, in the SEER database. We signed and adhered to the data use agreement for SEER radiation therapy and chemotherapy information to obtain the chemotherapy and radiation therapy data from SEER database. Specific site codes C30.0, C31.0, C31.1, C31.2, C31.3, C31.8, C31.9 were used to identify the specific location of the tumor in the nasal cavity or paranasal. Although modified Kadish staging was not available in the SEER database, we used SEER extent of disease, collaborative stage extension, historic stage, and primary site to deduce modified Kadish staging. Jethanamest et al. and Tajudeen et al. used this method of modified Kadish stage derivation for SEER studies pertaining to ENB [11, 18]. Extent of disease and collaborative staging extent codes for anatomic involvement of primary tumors were grouped and correlated with the appropriate modified Kadish stage as follows: confined to the nasal cavity (stage A), extension to the paranasal sinuses (stage B), extension beyond the nasal cavity and sinuses, including the cribriform plate and base of skull (stage C), and lymph node and distant metastases (stage D). We invited two experienced clinicians to derive the modified Kadish stage for the SEER cohort. When there are disagreements, they determined through consultation. We defined tumor differentiation grades I and II as low-grade tumors, and defined grades III and IV as high-grade tumors. The retrospective study followed the Helsinki Declaration (1964) and its later amendments or comparable ethical standards. No consent was required for the deidentified data, and no additional ethical approval processes were required for access to the database. Patient information was acquired by SEER*Stat software (version 8.3.6).

The inclusion criteria for the training cohort was as follows: 1. ENB with positive histological confirmation and not from an autopsy or death certificate; 2. active follow-up patients; and 3. known survival months after diagnosis and cause of death. The exclusion criteria for both the training cohort and the validation cohort were as follows: 1. unknown demographic information (age, race, sex); 2. unknown clinicopathological information (tumor grade and modified Kadish stage); 3. ENB was not the primary tumor if there were 2 or more; and 4. the follow-up time was less than 1 month. A total of 639 patients were excluded due to unknow demographic and clinicopathological information, or not first tumor, or follow up time less than 1 month after treatment. The flow diagram of training cohort data selection is shown in supplementary Figure 1. After applying the screen criteria, 225 patients were included in the final SEER cohort.

For the validation cohort, ENB patient data were collected from Sun Yat-sen University Cancer Center and The First Affiliated Hospital of Sun Yat-sen University in the same period. This study was approved by the institutional review boards of the First Affiliated Hospital, Sun Yat-sen University, Guangzhou, China ([2020]111). All inclusion criteria were identical to those used in the SEER cohort. A total of 96 patients with pathologic-proven ENBs were screened in this period, but 2 patients were excluded due to not first tumor and 7 patients were excluded because unknow demographic and clinicopathological information. After applying the screen criteria, 87 patients were included in the validation cohort. The Pearson’s chi-squared test was used to assess significance of clinicopathological and demographic characteristics.

### Modified Kadish classification and Hyams grading system

The modified Kadish classification was used to classify primary tumor extension: stage A is confined to nasal cavity, stage B extends into paranasal sinuses, stage C extends beyond the paranasal sinuses and stage D presents cervical lymph node involvement (supplementary Table 1). For training cohort, tumor grade based on record of the SEER data base, while for the validation cohort, the tumor grade criterion was the Hyams Grading system (supplementary Table 2) and reviewed by two trained pathologists.

### Definition of OS and survival analysis

OS was defined as the time from ENB diagnosis to the time of death or last follow-up. The OS length was calculated from the time of death for any cause or censoring. In this study, we used Kaplan-Meier analysis to calculate 3- and 5-year survival for covariates and used the log-rank test to determine statistical significance.

### Nomogram development

Based on the results of multivariate analysis, a nomogram model was formulated. All variables with significant differences at *p* < 0.05 in univariate analysis were included in the multivariate analysis. We used the Cox proportional hazards model for multivariate analyses. We used model coefficients to determine hazard ratios. Prognostic factors with a *p-*value < 0.05 in multivariate analysis were included in the nomogram. The optimal cut-off values of age group and the nomogram score for risk group stratification were calculated by X-tile 3.6.1 software (Yale University, New Haven, CT, USA).

### Nomogram validation

The nomogram’s predictive power was evaluated by the c-index for both of training cohort and validation cohort. We used the c-index to evaluate the predictive power of the nomogram for both cohorts. The c-index was used to quantify the difference between the prediction and the actual situation [19]. Values ranged from 0.5 (no discrimination) to 1.0 (complete discrimination). A larger C-index predicts a more accurate prediction of the prognosis. The agreement between predicted survival and the observed survival after bias correction was quantified by calibration curves of the nomogram for the 3-year and 5-year OS. Statistical analysis was conducted by R software version 3.5.2 (R Foundation for Statistical Computing, Vienna, Austria; www.R-project.org). All calculated *p* values were two-sided, and *p* < 0.05 was considered statistically significant.

## Results

### Clinicopathologic characteristics of patients and survival

The training cohort comprised 225 patients with ENB who were recruited between 1976 and 2016. The nomogram was based on the training cohort, and the median OS time was 48 months (1–155 months). In the training cohort, 122 (54.2%) patients were diagnosed at the age of 54 or younger. A total of 133 (59.1%) patients were males, and 92 (40.9%) were females. Additionally, 129 patients had a low-risk tumor grade, accounting for 57.3% of the total, and 96 (or 42.7%) had a high-risk tumor grade. A total of 60.9% of the patients were diagnosed with stage C disease. Most of the patients in training cohort had received surgery (90.2%) and radiotherapy treatment (68.0%), while most of the patients had no chemotherapy treatment or had no information about chemotherapy (64.9%). In terms of treatment options, due to the limitation of the SEER database, the sequence of chemotherapy with surgery and radiotherapy was unknown.

For the validation cohort, we studied 87 consecutive patients in the same period, and the median OS time was 29 months (1–208 months). Fifty-eight (66.7%) patients were males and 29 (33.3%) patients were females in the validation cohort. The most common age at diagnosis of these patients with ENB was ≤54 (70.1%). With regard to tumor stage, modified Kadish C stage (42.5%) was most frequent, followed by B stage (29.9%), D stage (26.4%) and A stage (1.1%). The majority of patients received radiotherapy (66.7%). Only 41.4% of patients in the Chinese cohort had received surgical treatment (Table 1).

**Table 1:** Demographic and clinicopathological characteristics of patients with ENB

Characteristics	Training cohort (*N* = 225) (%)	Validation cohort (*N* = 87) (%)	*P* value
Gender (%)			**0.136**
Female	92 (40.9)	29 (33.3)	
Male	133 (59.1)	58 (66.7)	
Age (%)			**0.034**
< =54	122 (54.2)	61 (70.1)	
55–69	73 (32.4)	20 (23.0)	
> =70	30 (13.3)	6 (6.9)	
Tumor Grade (%)			**0.304**
Low	129 (57.3)	56 (64.4)	
High	96 (42.7)	31 (35.6)	
Modified Kadish(%)			**0.002**
A	49 (21.8)	1 (1.1)	
B	13 (5.8)	26(29.9)	
C	137 (60.9)	37 (42.5)	
D	26 (11.6)	23 (26.4)	
Chemotherapy(%)			**0.005**
N	146 (64.9)	42 (48.3)	
Y	79 (35.1)	45 (51.7)	
Radiotherapy(%)			**0.461**
N	72 (32.0)	29 (33.3)	
Y	153 (68.0)	58 (66.7)	
Surgery(%)			**< 0.001**
N	22 (9.8)	51 (58.6)	
Y	203 (90.2)	36 (41.4)	

*N* No/Unknown, *Y* Yes

**Fig. 1:**
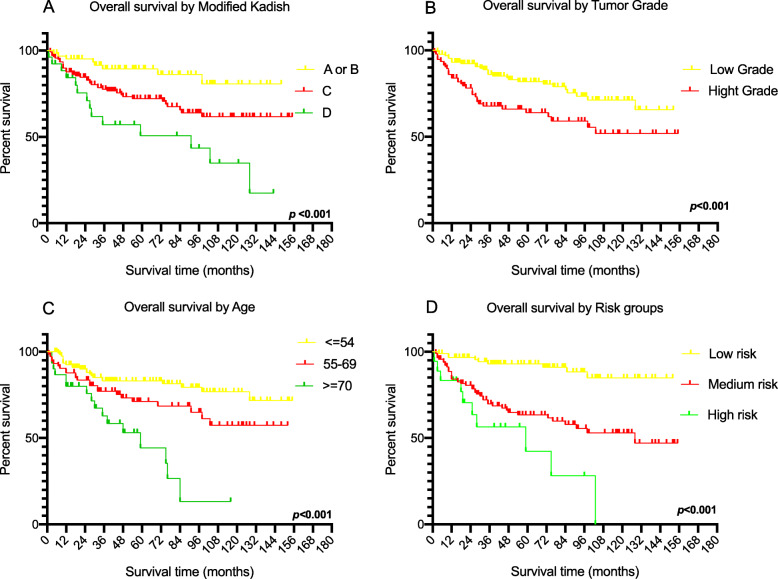
Kaplan-Meier survival curves for the factors of the primary cohort **a** the diference of the overall survival in Modified Kadish stage; **b** the diference of the overall survival in Tumor Grade; **c** the diference of the overall survival in Age. **d** the diference of the overall survival in risk group

Patient characteristics and tumor characteristics were assessed by Kaplan-Meier survival analysis. The modified Kadish A or B group had the highest 5-year OS rate (89.4%). The C group and D group represented 72.1 and 50.7%, respectively (Fig. 1a). In terms of the tumor differentiation grade characteristics, patients in the high-grade group had a comparatively lower OS rate, reaching 63.9%, while in the low-grade group, the 5-year OS rate was 81.8% (Fig. 1b). The 5-year OS rates for training cohort patients younger than 55 years old, 55–69 years old and older than 70 were 83.1, 71.2 and 44.2%, respectively (Fig. 1c). The 5-year OS of the low-risk group was 93.0%, followed by the medium-risk group (63.4%) and the high-risk group (28.3%) (Fig. 1d).

### Independent prognostic factors of OS

Univariate analyses have demonstrated that modified Kadish stage, gender, tumor differentiation grade, age at diagnosis, chemotherapy, and surgery are associated with OS. We included all of the above prognostic factors with *p* < 0.05 in the multivariate analysis, and multivariate analysis showed that modified Kadish stage, age at diagnosis, and tumor differentiation grade were independent risk factors for patients with ENB. The detailed results of the multivariate analysis are presented in Table 2.

**Table 2:** Cox Proportional Hazards Regression Analyses of overall survival for ENB patients in the training Cohort

Variable	Univariate analysis		Multivariate analysis	
	HR (95%)	*P*	HR (95%)	*P*
Sex (%)	Ref			
Female	1.392 (0.826–2.344)	0.214	NI	
Male			NI	
Age(%)				
< =54	Ref		Ref	
55–69	1.787 (1.002–3.186)	0.049	1.726 (0.962–3.097)	0.067
> =70	4.131 (2.160–7.900)	< 0.001	3.773 (1.950–7.505)	< 0.001
Tumor Grade(%)				
Low	Ref		Ref	
High	2.240 (1.354–3.707)	< 0.001	1.991 (1.151–3.444)	0.014
Modified Kadish				
A or B	Ref		Ref	
C	2.536 (1.187–5.42)	0.016	1.950 (0.892–4.263)	0.094
D	5.246 (2.199–12.51)	< 0.001	2.797 (1.057–7.401)	0.038
Chemotherapy				
N	Ref		Ref	
Y	1.857 (1.126–3.061)	0.015	1.161 (0.635–2.122)	0.628
Radiotherapy				
N	Ref		NI	
Y	0.725 (0.435–1.209)	0.218	NI	
Surgery				
N	Ref		Ref	
Y	0.407(0.217–0.764)	0.005	0.729 (0.362–1.470)	0.378

*N* No/Unknown, *Y* Yes, *NI* Not include, *Ref* Reference, *HR* Hazard Ratio

### Nomogram construction and risk stratification

**Fig. 2:**
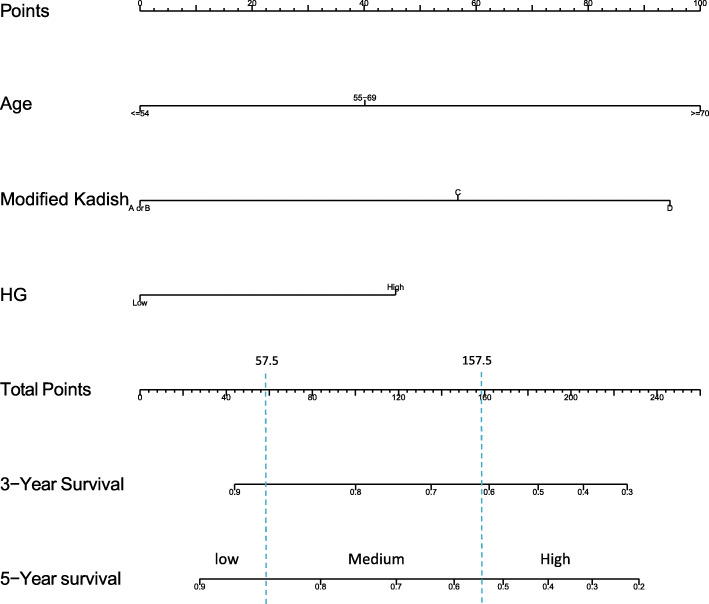
Nomograms developed for 3- and 5-year prediction of overall survival for esthesioneuroblastoma patients. Notes: Drawing the vertical line between points scale and each variable to acquire points of each variable. According to the total points, predicted survival probability was calculated by drawing a vertical line from Total Points scale to overall survival scale

In the Cox model, modified Kadish stage, tumor differentiation grade, and age at diagnosis were independent prognostic factors revealed by multivariate analyses. Modified Kadish stage, tumor differentiation grade, and age at diagnosis were used to develop the nomogram for estimating 3- and 5-year OS (Fig. 2). To use a nomogram, lines are drawn to score the prognostic variables on the top point scale for an individual patient. The number of points received for each variable value and the score for each prognostic variable on the point scale are added together. The sum of scores is on the total point axis, and one line is drawn to the survival axis to convert to a 3- or 5-years probability.

Patients were subdivided into a low-risk group (0 ≤ score ≤ 57.5), an intermediate-risk group (scoring 57.5 < score < 157.5) and a high-risk group (157.5 ≤ score ≤ 300).

### Nomogram validation

**Fig. 3:**
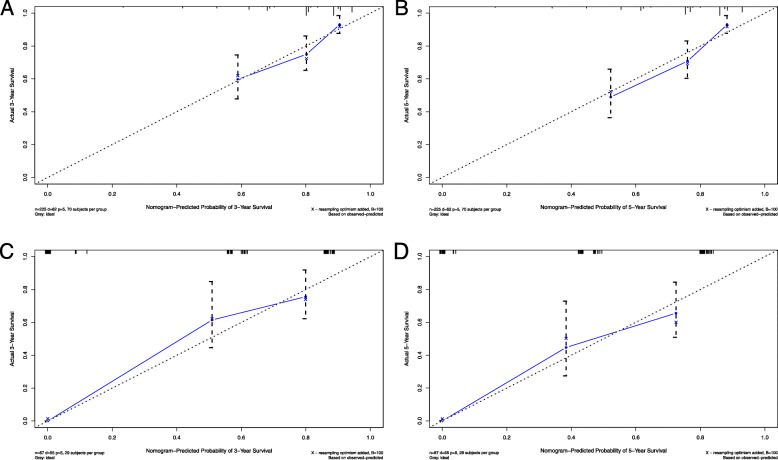
Calibration plots in the primary cohort (**a** and **b**) and the validation cohort (**c** and **d**) for predicting patient survival at 3 years and 5 years. Nomogram-predicted probability of overall survival is plotted on the *x*-axis; actual overall survival is plotted on the *y*-axis, and the dashed line (the 45° line) represents the ideal nomogram plot

In this study, we performed both internal and external validation of the nomogram. The plotted calibration curves corresponded to the ideal plot (the 45°line), which revealed a favorable agreement on the nomogram estimation and actual observation regarding the probability of 3-year and 5-year survival (Fig. 3a, b, c, d). In the training cohort, the model showed a high accuracy with a c-index of 0.737 (95% CI, 0.709 to 0.765) which was higher than the modified Kadish staging system, at 0.614 (95%CI, 0.579 to 0.649). In the validation cohort, the nomogram prediction was 0.791 (95% CI, 0.767 to 0.815) was also higher than for the modified Kadish staging system prediction (0.674, 95% CI, 0.643–0.705). These results suggest that the nomogram was reasonably accurate, repeatable and had a better accuracy in predicting OS than the modified Kadish staging system.

## Discussion

Recently, several disease centers have published their own treatment experience, but each single-center study of the disease was generally limited by the sample size, which had a certain impact on the accuracy of the results. Modified Kadish staging system was the most widely used ENB staging system [10, 20]. Recent studies have shown that the survival of patients with ENB was significantly associated with pathologic grade and age [18, 21]. To the best of our knowledge, this is the first study to use the nomogram model to combine age, pathologic grade, and modified Kadish staging systems to predict the prognosis of patients with ENB.

Although surgery and chemotherapy were associated with patient outcomes in univariate analysis, they were not prognostic independent predictors in multivariate analysis. Patients receiving chemotherapy often have large local tumors or distant metastases [6]. For patients with locally advanced tumors, chemotherapy decreased the risks of systemic failure by acting on systemic micrometastasis [22]. For patients with distant metastases who did not undergo radical surgery, chemotherapy may be a suitable treatment and control the lesions. Compared with the SEER cohort, the Chinese cohort had relatively fewer patients undergoing surgery and a higher proportion of patients receiving chemotherapy. One reason for this phenomenon was that the proportion of distant metastasis in the Chinese cohort was significantly higher than that in the Western cohort (26.4% vs. 11.6). This may be due to bias caused by too small a sample size. Another potential reason was that Sun Yat-sen University Cancer Center and The First Affiliated Hospital of Sun Yat-sen University were two famous hospitals in China, a bias toward more advanced disease among those referred to these two hospitals. Last but not least, the lack of ascertainment in SEER and the inevitable selection bias might be weighted towards a surgical group.

The role of age in ENB is still controversial and unclear. In this study, the best cutoff values of 54 and 70 were calculated by X-tile [23], and the prognosis was the best in the group of patients younger than 54 years old. Although these patients all received the same treatment strategy, this study still showed different survival trends in three groups. Yin et al. showed that patients older than 60 years of age had a worse prognosis [21]. Previous studies have shown that young patients with ENB have more aggressive disease, but these patients are sensitive to chemotherapy and can achieve good results through a combination of chemotherapy and radiotherapy [24]. We recommend that young, locally advanced patients enter Multi-Disciplinary Therapy Meeting (MDT) to discuss and determine treatment options.

The standard for pathological grading of ENB is the Hyams standard. At present, some studies have reported that pathological graded survival was significantly correlated and was an independent predictor of survival in patients with ENB [11, 25–27]. For Chinese cohort, the tumor differentiation criterion based on the Hyams Grading System while the SEER cohort used the tumor differentiation grading scheme. The indicators for evaluating cell differentiation included mitotic index and nuclear polymorphism, which were also part of the Hyams scoring system. Limited by the SEER database, it could not provide Hyams grading information, but the impact on nomogram might be slight. Tajudeen et al. considered that tumor differentiation grading scheme roughly corresponded to the Hyams grading scale [11]. Significant differences in survival can be seen in the pathological graded polarization of ENB, and high-grade pathological differentiation grades tend to have a worse prognosis [26]. In this study, we defined grade I and grade II tumors as low-grade tumors and defined grade III and grade IV tumors as high-grade tumors. In these two groups of patients, we observed significant differences in both training cohort and validation cohort, while in multivariate analysis, high-grade tumors were risk factors for prognosis. For SEER grading scheme may not be interpreted as a true Hyams grade, but it roughly corresponds to the Hymas grading scale. The bias caused by this method requires a large sample size cohort containing Hyams grading information as training cohort to reconstruct a nomogram. However, due to the rarity of ENB, the SEER database was the largest cohort that could be obtained, and variability was minimized by grouping patients into low-grade and high-grade tumor groups.

A number of studies evaluated the predictive power of the modified Kadish staging system [6, 20, 28]. Although it was partly confirmed that the modified Kadish staging system can effectively predict the prognosis of patients, some of them did not confirm its predictive efficacy. The reason for this phenomenon was the lack of sample size, selection bias, or a defect in the modified Kadish staging system itself. In the present study, we did not find statistically significant differences in survival between modified Kadish A and B, either in the SEER cohort or in the Chinese cohort. This was consistent with the conclusions of some previous studies [28]. Therefore, to improve statistical performance, stage A and stage B ENB patients were combined together. The prognosis of these patients was significantly better than that of patients with stage C and stage D disease.

The prognostic significance of clinical staging and pathologic grading were perhaps confounded often by each other [6, 25]. These factors explained the limitations of using pathological grading and clinical grading alone to some extent. The use of the modified Kadish staging system and pathological grading system was not sufficiently accurate. We established a nomogram to predict the prognosis of patients. Based on the Cox regression risk model, the model calculated the likelihood of 3-year and 5-year survival based on the patient’s age, clinical stage and tumor pathologic grade. Clinical application was simple and convenient. Here, we demonstrated a nomogram application example based on the calculation of the nomogram. The patient was a 55-year-old male who was diagnosed with clinical stage C in 2012. The tumor pathologic differentiation grade was a low-risk group (grade I well differentiated), and the patient underwent both surgery and radiotherapy. According to the nomogram, the measured probability of 3-year survival was about 83%, and the 5-year survival was about 80%. This patient was in the low-risk group. When this new method of evaluating prognosis is extended to patients in the non-SEER cohort, we recommended using this nomogram after the validation step. It can reduce the bias caused by selection and regional differences in medical levels. It may be necessary to build a nomogram with the data of the non-SEER population, and then determine the cut-off value of the risk stratification according to the actual situation of the population by using our methods.

The c-indexes for internal and external validation were 0.737 (95% CI, 0.709 to 0.765) and 0.791 (95% CI, 0.767 to 0.815), respectively, which showed that the present nomogram was a repeatable and accurate prognostic tool for predicting 3- and 5-year OS in patients. However, some of ENB patients could have a long naturel history, OS may not be the most relevant endpoint. Quality of life for ENB patients was one of dominant components of the treatment evaluation. Thus, DFS may be more relevant than OS, but SEER database only provides OS and disease-specific survival as primary endpoints. Further improvement of our nomogram by using patient series with data for quality of life and DFS is needed. Nonetheless, this nomogram could act as a tool to select high-risk patients and make individualized treatment and follow-up schedules.

There were several limitations in our study. First, this was a retrospective analysis study that inevitably had a selective bias. One of the enrollment criteria used positive histology only, which resulted in some patients who did not receive surgery and lacked pathological data were excluded from the cohort. A total of 599 patients were excluded due to unknow demographic and clinicopathological information and 279 out of 599 patients (46.6%) had no surgery performed. The proportion of excluded patients who received surgery was lower than the proportion of enrolled patients (53.4% vs. 90.2%). This might increase the proportion of patients in the SEER cohort who received surgery. Second, the SEER database did not provide detailed chemotherapy information. In this study, we were unable to confirm information about the course of treatment for patients with chemotherapy, which may lead to bias in the treatment results. In addition, the detailed radiation therapy data are not provided, and it was hard to evaluate the treatment impact on the SEER cohort’s patients. Third, the SEER database did not provide the patient’s surgical methods, so it was impossible to make comparisons on the influence of the surgical approach. Finally, the SEER database did not provide patients’ information about modified Kadish stage and Hyams grade. Modified Kadish staging transformation depended on the accurateness of SEER data and its coding system. Nonetheless, the results were still novel, we successful provided insight into the utility of the nomogram and to verify the repeatability and practicability of the nomogram in validation cohort.

## Conclusion

The study explored a nomogram based on clinical characteristics, treatment and pathological variables in predicting OS among patients with ENB. The present study identified modified Kadish staging system, tumor differentiation grade, and age at diagnosis as independent prognostic variables for the OS rates of patients with ENB. We used a nomogram to calculate the 3-year and 5-year growth probability and stratified patients into three risk groups. The nomogram provided the risk group information and identified mortality risk and can serve as a reference for a more reasonable follow-up plan.

## Supplementary information

**Additional file 1: Figure S1.** Flowchart of included population in this study.

**Additional file 2.**

## Data Availability

The datasets used and/or analysed during the current study are available from the corresponding author on reasonable request.

## Abbreviations

ENB: Esthesioneuroblastoma
OS: Overall Survival
SEER: Surveillance, Epidemiology, and End Results
HR: Hazard ratios
CI: Confidence interval
C-index: Concordance index
DFS: Progression-free survival
MRI: Magnetic resonance imaging
CT: Computed tomography
MDT: Multi-Disciplinary Therapy Meeting
