# Supplementary figures and images for: A novel nomogram to predict the overall survival in esthesinoeroblastoma
Source: BMC Cancer. 2020 Oct 14;20:993. doi: 10.1186/s12885-020-07435-7 (PMC7556920; doi:10.1186/s12885-020-07435-7)

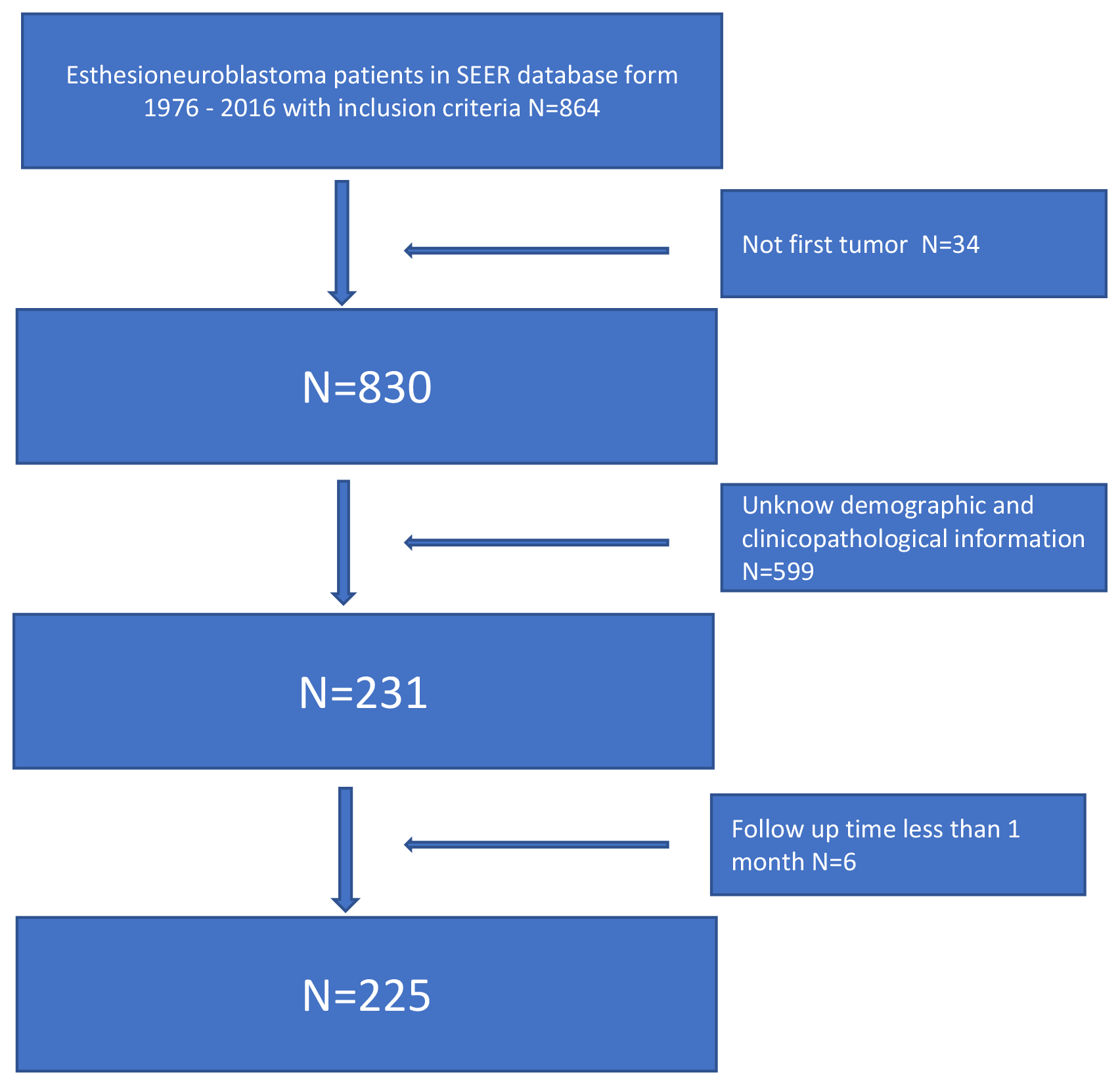

Supplement: Supplementary file 1 — Additional file 1: Figure S1. Flowchart of included population in this study. [file 12885_2020_7435_MOESM1_ESM.tif]
