# Supplementary material for: A novel nomogram to predict the overall survival in esthesinoeroblastoma
Source: BMC Cancer. 2020 Oct 14;20:993. doi: 10.1186/s12885-020-07435-7 (PMC7556920; doi:10.1186/s12885-020-07435-7)
Supplement: Supplementary file 2 — Additional file 2. [file 12885_2020_7435_MOESM2_ESM.docx]

**Supplementary tables**

**Table 1** **Modified Kadish staging Classification for Esthesioneuroblasoma [10]**

| Staging | Description |
| --- | --- |
| A | Tumor confined to the nasal cavity |
| B | Tumor extension to the paranasal sinuses |
| C | Tumor beyond the nasal cavity and paranasal sinuses, including involvement of the cribriform plate, base of the skull, intracranial cavity, and/or orbit |
| D | Tumor with metastases to cervical lymph nodes and/or distant sites |

**Table 2** **Histopathological grading, according to Hyams [7]**

| Grade | Lobular architecture preservation | Mitotic index | Nuclear polymorphism | Fibrillary matrix | Rosettes | Necrosis |
| --- | --- | --- | --- | --- | --- | --- |
| Ⅰ | + | none | none | prominent | HW rosettes | none |
| Ⅱ | + | low | moderate | present | HW rosettes | none |
| Ⅲ | +/- | moderate | prominent | low | FW rosettes | rare |
| Ⅴ | +/- | high | marked | absent | none | frequent |

+ present; +/- , may be present or absent; HW, Homer Wright; FW, Flexner-Wintersteiner rosettes
